# *In Vitro* Antibacterial Experiment of Fuzheng Jiedu Huayu Decoction Against Multidrug-Resistant *Pseudomonas aeruginosa*

**DOI:** 10.3389/fphar.2019.01682

**Published:** 2020-02-12

**Authors:** Hongri Xu, Chang Liu, Meng Li, Chengxiang Wang, Guoxing Liu, Honghong Wang, Jie Ma, Lei Li, Meng Chen, Miao Cheng, Xingwei Yao, Ying Lin, Shitong Zhao, Yuting Wang, Mingzhe Wang

**Affiliations:** ^1^ Emergency Department, Beijing University of Chinese Medicine (BUCM) Third Affiliated Hospital, Beijing, China; ^2^ Respiratory Department, BUCM Third Affiliated Hospital, Beijing, China; ^3^ Respiratory Department, ShaanXi Traditional Chinese Medicine (TCM) Hospital, Xi'an, China; ^4^ Respiratory Department, Beijing Hospital of Integrated Traditional Chinese and Western Medicine, Beijing, China; ^5^ Institute of Industrial and Consumer Product Safety, China Academy of Inspection and Quarantine, Beijing, China; ^6^ Respiratory Department, Dongzhimen Hospital Affiliated to BUCM, Beijing, China; ^7^ Clinical Laboratory, Dongzhimen Hospital Affiliated to BUCM, Beijing, China; ^8^ Respiratory Department, China–Japan Friendship Hospital, Beijing, China

**Keywords:** growth curve, Fuzheng Jiedu Huayu decoction, multidrug-resistant *Pseudomonas aeruginosa*, *in vitro* antibacterial, drug sensitivity test

## Abstract

**Objective:**

Drawing a growth curve of multidrug-resistant *Pseudomonas aeruginosa* (MDR-PA) provides a foundation for susceptibility testing. By observing *in vitro* antibacterial activity and ultrastructure cthanges on MDR-PA of the effective components in the drug-containing serum of rats after the administration of Fuzheng Jiedu Huayu decoction (FJHD), we evaluated the inhibition and direct destruction effect of bacteria by TCM alone or combined with antibiotics.

**Methods:**

The absorbance values of MDR-PA were determined at different detection time points, and a growth curve was drawn. After gavage with FJHD, drug-containing serum was collected from the rats. Using Imipenem/cilastatin sodium as the positive drug control, the *in vitro* antibacterial potency of FJHD and its drug-containing serum alone or in combination with antibiotics against MDR-PA was observed. The ultrastructural changes of MDR-PA treated by FJHD combined with antibiotics were observed by transmission electron microscopy.

**Results:**

Growth of the experimental strain manifested a lag phase in the first 1–4 h, an exponential growth phase at 5–20 h, and a plateau phase after 20 h. The best detection time during the susceptibility test was 16–20 h. The minimum inhibitory concentration (MIC) value of the FJHD extract group was 0.2 g/mL. The MIC value of the pure Imipenem/cilastatin sodium group was 16 μg/mL. The MIC values of Imipenem/cilastatin sodium + blank serum, 0.5-, 1-, and 2-fold drug-containing serum groups were all 16 μg/mL. The MIC values of Imipenem/cilastatin sodium + 4- and 8-fold drug-containing serum groups were both 8 μg/mL. By observation under a transmission electron microscope, Imipenem/cilastatin sodium + 0.5-, 1-, and 2-fold drug-containing serum groups showed bacterial structural damage. The degree of bacterial destruction was more obvious and the quantity of damaged bacteria was increased in the Imipenem/cilastatin sodium + 4- and 8-fold drug-containing serum groups.

**Conclusion:**

Drawing the growth curve of the experimental strain had high application value for ensuring the accuracy of the drug sensitivity test results. TCM combined with antibiotics could enhance the antibacterial and direct destruction effect of bacteria *in vitro*, thereby inhibiting bacterial resistance to a certain extent.

## Introduction

Due to the extensive application of antibacterial drugs worldwide, the problem of bacterial resistance is becoming increasingly serious. The pressure to choose antimicrobial agents is increasing, and the efficacy of refractory infectious diseases caused by drug-resistant pathogens is often not satisfactory (Mullar and O’Neill, 2016). Elderly patients, long-term bedridden patients, patients using glucocorticoids, patients with blood disorders, and cancer patients tend to have lower immune function, poorer disease-resistant ability, and increased susceptibility to drug-resistant pathogens. During the long-term treatment of infectious diseases in clinical work and research, our research group found that patients with basic diseases, such as chronic obstructive pulmonary disease, cardiac insufficiency, stroke, Parkinson's disease, diabetes, and malnutrition, and elderly patients with repeated use of antibacterial drugs, are more prone to drug-resistant bacteria infection (Xu et al., 2011). One of the most common infection sites is the lung, and treatment effects are often poor. Antibiotic-resistant pneumonia in the elderly belongs to the category of “wind-warmth lung heat disease” in Traditional Chinese Medicine (TCM). “Deficiency of vital *qi*, toxic heat flaming, and toxin-blood stasis” is present in drug-resistant pneumonia in the elderly, which is the basic TCM pathogenesis of this condition. In a previous multicenter, prospective, double-blind, parallel, randomized controlled trial (Xu et al., 2018), we found that Fuzheng Jiedu Huayu decoction (FJHD) full active ingredient extract formula granule, based on the above basic TCM pathogenesis of pneumonia in the elderly, significantly improved the curative effects of patients after a 2-week treatment course, ameliorating expectoration, and promoting the absorption of pneumonia lesions. At the same time, we found that pneumonia in the elderly with a course of more than 2 weeks was often caused by drug-resistant pathogens, especially *Pseudomonas aeruginosa* (PA). FJHD full active ingredient extract formula granules showed effectiveness in the treatment of elderly drug-resistant pneumonia in previous clinical research (Xu et al., 2018). To further explore and reveal the mechanism of FJHD full active ingredient extract formula granules for treating pneumonia caused by drug-resistant bacteria infection, the antibiotic Imipenem/cilastatin sodium, which is commonly used in clinical practice and has strong antibacterial effect, was used as the positive drug control. After gavage with FJHD, rat drug-containing serum was collected to conduct *in vitro* antibacterial experiments on a kind of Gram-negative bacteria, multidrug-resistant *P. aeruginosa* (MDR-PA), which often causes drug-resistant pneumonia in the elderly, to observe the antibacterial effect of the active components in the serum of rats after gastric gavage by FJHD, and to observe the ultrastructural changes of MDR-PA after treatment with FJHD, exploring the effects of FJHD on the MDR-PA ultrastructure. The growth curve of MDR-PA was drawn, and the best detection time point was determined to ensure the accuracy of the drug sensitivity test results.

## Materials and Methods

### Drugs

**Figure 1:**
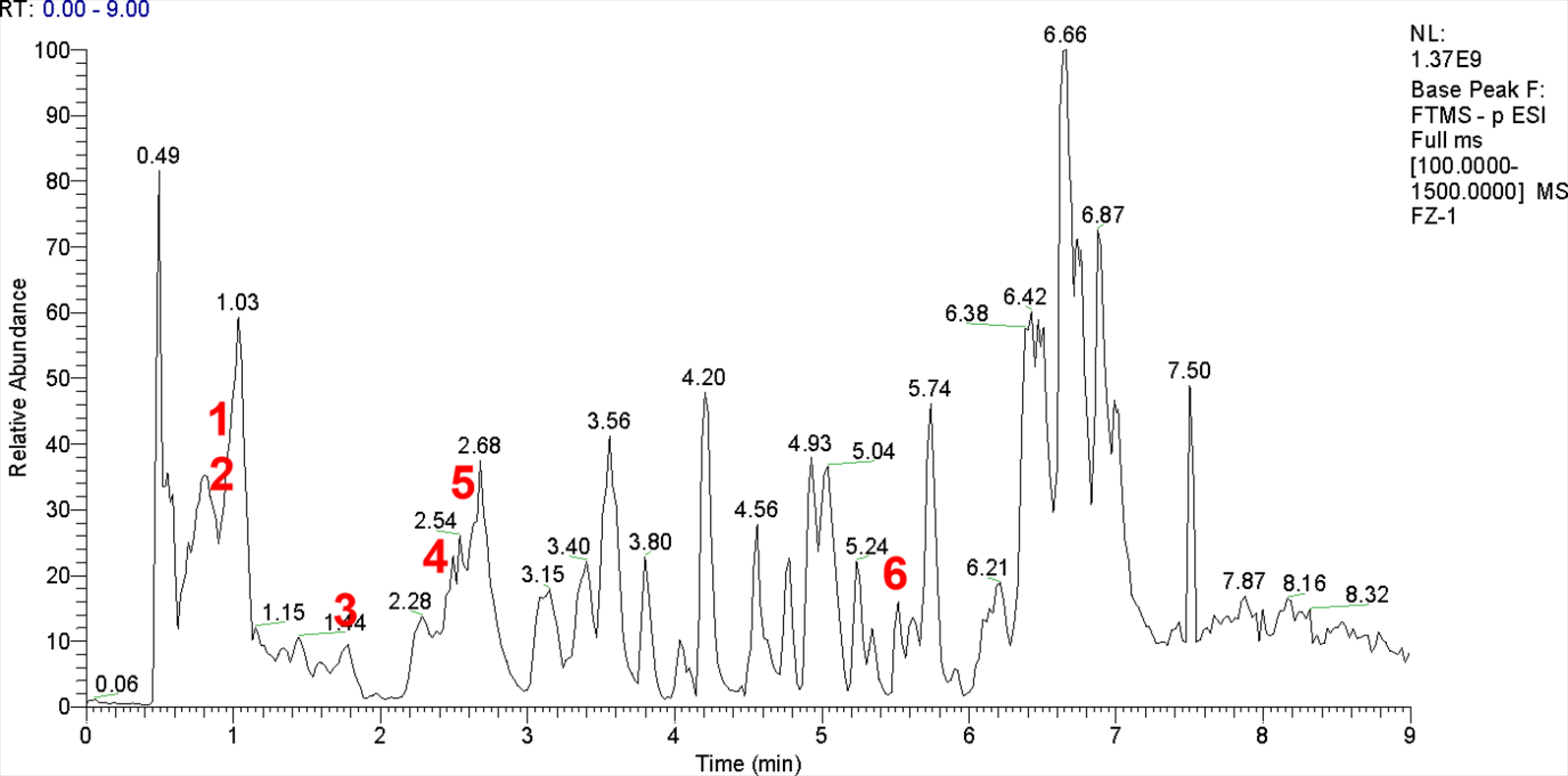
UHPLC-Q-Orbitrap HRMS chemical fingerprint of FJHD. Forsythiaside A(**1**), Paeoniflorin(**2**), β-ecdysterone(**3**), Forsythin(**4**), Baicalin(**5**), Ginsenoside Rb1(**6**).

FJHD full active ingredient extract formula granules were purchased from Beijing Tcmages Pharmaceutical Co., Ltd. (Beijing, China). FJHD consists of eight herbs: scutellariae radix (Batch No: 15017541), forsythiae fructus (Batch No: 15020481), echinopsis radix (Batch No: 14019491), paeoniae radix alba (Batch No: 15012061), trichosanthis radix (Batch No: 15020191), panacis quinquefolii radix (Batch No: 15010202), patriniae radix (Batch No: 15014241), coicis semen (Batch No: 15018821), and the mixed proportion of respective herbs is illustrated in **Table 1**. A method of simulated family decoction was used to extract the herb, and then the extracts were concentrated and dried to form granules (Beijing Tcmages Pharmaceutical Co., Ltd., 2013). This process was performed according to Good Manufacturing Practice for Drugs (Chinese FDA, version 2010) to guarantee quality control. Furthermore, the obtained products were re-identified by the School of Chinese Materia Medica, Beijing University of Chinese Medicine (Beijing, China). All of the samples were deposited in the herbarium of Beijing Tcmages Pharmaceutical Co., Ltd. (Beijing, China) with voucher number CP1-3-7-10 for scutellariae radix, CP2-2-6-12 for forsythiae fructus, CD15-7-2-1 for echinopsis radix, CP1-6-1-10 for paeoniae radix alba, CP3-8-2-5 for trichosanthis radix, CD1-7-4-4 for panacis quinquefolii radix, CD3-4-4-13 for patriniae radix, and CP4-8-2-1 for coicis semen. The quality of the herbs and herbal extracts were consistent with the standards of Chinese Pharmacopoeia (2015). The chemical fingerprint (ultra- high performance liquid chromatography- Q Exactive hybrid quadrupole-orbitrap high resolution accurate mass spectrometry [UHPLC-Q-Orbitrap HRMS]) of FJHD was analyzed (see **Figure 1**) and the detailed methods of UHPLC-Q-Orbitrap HRMS are provided in the **Supplementary Data**.

**Table 1:** Composition of FJHD.

Botanical name	Herbal name	Chinese name	Dosage	Voucher Number
*Scutellaria baicalensis* Georgi	Scutellariae radix	Huang Qin	15g	CP1-3-7-10
*Forsythia suspensa* (Thunb.) Vahl	Forsythiae fructus	Lian Qiao	15g	CP2-2-6-12
*Echinops latifolius* Tausch	Echinopsis radix	Lou Lu	15g	CD15-7-2-1
*Paeonia lactiflora* Pall.	Paeoniae radix alba	Chi Shao	15g	CP1-6-1-10
*Trichosanthes kirilowii* Maxim.	Trichosanthis radix	Gua Lou	30g	CP3-8-2-5
*Panax quinquefolius* L.	Panacis quinquefolii radix	Xi Yang Shen	6g	CD1-7-4-4
*Patrinia scabiosifolia* Link	Patriniae radix	Bai Jiang Cao	30g	CD3-4-4-13
*Coix lacryma-jobi* var. *ma-yuen* (Rom. Caill.) Stapf	Coicis semen	Sheng Yi Mi	30g	CP4-8-2-1

Imipenem/cilastatin sodium(Product name: Tienam, Serial Number: Product with code number approved by SFDA J20130123, Merck Sharp & Dohme Corp., Kenilworth, NJ, USA.) was purchased from the Pharmacy Department, Dongzhimen Hospital Affiliated to BUCM, and formulated into a solution with a concentration of 1,024 μg/mL using phosphate-buffered saline (PBS), as a positive drug control.

### Animals

Eighty male SPF grade Sprague-Dawley (SD) rats, 70–76 days old, weighing 350–460 g, were purchased from Beijing Weitong Lihua Experimental Animal Technology Co., Ltd. (Animal Batch No: 11400700120906; Beijing, China).

This study was conducted in accordance with the recommendations of the Guide for Care and Use of Laboratory Animals published by the U.S. National Research Council [National Research Council (US) Institute for Labaratory Animal Research, 1996], and Beijing University of Chinese Medicine Medical and Experimental Animal Ethics Committee. The protocol was approved by the Beijing University of Chinese Medicine Medical and Experimental Animal Ethics Committee.

### Strain

MDR-PA clinical strain (No. 1508032) was purchased from the bacteria room of the Clinical Laboratory, Dongzhimen Hospital Affiliated to BUCM.

### Reagents and Instruments

In this study, we used the following materials: MH agar medium, MacConkey medium, and nutritional broth were purchased from Thermo Fisher Scientific Chemicals Co., Ltd. (Waltham, MA, USA). Nutrition broth was prepared with deionized water and autoclaved.

The following instruments were employed: constant temperature and humidity incubator (Cat. No: LHS-250HC-11; Shanghai Yiheng Scientific Instrument Co., Ltd., Shanghai, China), constant temperature oscillator (Cat. No: DDHZ-300; Suzhou Peiying Experimental Equipment Co., Ltd., Jiangsu, China), pressure steam sterilizer (Cat. No: MSG.N 180L; Shandong Weigao Group Medical Polymer Products Co., Ltd., Weihai, China), low speed centrifuge (Beijing Baiyang Medical Devices Co., Ltd., Beijing, China), precision electronic balance (Cat. No: BS124S; Beijing Sartorius Instrument System Co., Ltd., Beijing, China), and microplate reader (Cat. No: Multiskan MK3; Thermo Fisher Scientific Chemicals Co., Ltd.).

### Grouping and Administration

The rats were divided into blank group and FJHD groups by random grouping using SPSS 19.0 at concentrations of 0.5-, 1-, 2-, 4-, and 8-fold of the equivalent dose, and formulated into a Chinese medicine suspension with concentrations of 0.5, 1, 2, 4, and 8 g/mL. There were 10 experimental rats in each group. The blank group was given normal saline. The FJHD groups were given FJHD full active ingredient extract formula granules. Each group was administered twice a day for 5 days.

### Sampling

At 1 h after the last gavage, SD rats were anesthetized with chloral hydrate. With a sterilized technique, blood was collected through their abdominal aorta and centrifuged at 1,610 × g for 10 min to separate the drug-containing serum. The same group of serum was mixed with each other. Then the serum was inactivated at 56°C for 30 min in water bath, filtered through a 0.22-μm pore size filter, dispensed into a cryotube, and stored in a –70°C refrigerator. Mixing, filtration, and dispensing were all performed in a biosafety cabinet.

### Strain Activation and Bacterial Suspension Preparation

A small amount of MDR-PA cells (clinical strain No. 1508032) cryopreserved in an –80°C refrigerator were inoculated on MacConkey agar medium and cultured at 35°C for 18–24 h to activate the strain. The next day, the MDR-PA bacterial solution was adjusted to a concentration of 1 × 10^7^ cfu/mL by a turbidimetric method with sterile physiological saline.

### Determination of Growth Curve of MDR-PA by Microplate Reader

Four 50-mL sterile centrifuge tubes were taken, followed by the addition of 20 mL broth. To tubes 1–3, 1 mL prepared bacterial solution (the concentration of the bacterial solution in each tube was 5 × 10^5^ cfu/mL) was added. Tubes 1–3 were parallel test tubes, and the 4^th^ tube was a blank control tube with no bacterial fluid, as a negative control. The tubes were placed in a constant temperature oscillator for shaking culture (temperature: 37°C, rotation speed: 250 rpm). In accordance with relevant literature (Guo et al., 2011; Kong et al., 2014), wavelengths of 200 nm to 700 nm were scanned with 5 nm intervals by the full-band spectral scanning method to determine the best detection wavelength of MDR-PA. According to the literature (Guo et al., 2011; Kong et al., 2014) and test results, the optimum detection wavelength of MDR-PA absorbance was finally selected at 570 nm. After 1 h of bacterial culture, the OD570_nm_ value of each centrifuge tube was measured on a microplate reader. A volume of 200 μL from each centrifuge tube was added to a 96-well plate. Three replicate wells were made at each time point, and the OD570 _nm_ value of each centrifuge tube was measured every 1 h until 24 h. The OD570_nm_ value was analyzed by statistical software, and a standard growth curve was drawn to determine the optimal culture time in an *in vitro* bacteriostatic test.

### The Minimum Inhibitory Concentration Value Determination of the Tested Chinese Medicine by the Test Tube Dilution Method

In this experiment, the minimum inhibitory concentration (MIC) values of different doses of Chinese herbal extracts and drug-containing serum on MDR-PA were determined by tube dilution method (serial dilution method and double dilution method) (Zhou et al., 2005; Zhou et al., 2012). All of the aseptic procedures were performed in a biosafety cabinet.

#### MIC Value Determination of Simple Chinese Medicine Extract and Simple Drug-Containing Serum

Nine sterile tubes were taken from each group; 1 mL broth was added to tubes 1–7 and 2 mL was added to tubes 8–9. Volumes of 1, 0.8, 0.6, 0.4, 0.3, 0.2, and 0.1 mL Chinese herbal extract/drug-containing serum were added to tubes 1–7, and broth was added to a total volume of 2 mL in tubes 2–7. The 8^th^ tube served as the negative control and did not have bacterial liquid. The 9^th^ tube served as the model control and did not have Chinese herbal extract (or drug-containing serum). The prepared 0.5 Mc concentration solution was diluted by 10-fold, corresponding to a bacterial liquid concentration of 1.5 × 10^7^ cfu/mL, followed by the addition of 0.1 mL to each tube. After shaking for 30 s, the MDR-PA cells were cultured in a 35°C incubator, and growth of the bacteria was observed after 24 h. The composition of the test tube system is shown in **Table 2**.

**Table 2:** The Composition of simple Chinese medicine/drug-containing serum group test tube system.

System composition	Test tube number								
	1	2	3	4	5	6	7	8	9
Chinese medicine/drug-containing serum(ml)	1	0.8	0.6	0.4	0.3	0.2	0.1	0	0
Broth(ml)	1	1	1	1	1	1	1	2	2
Added broth(ml)	0	0.2	0.4	0.6	0.7	0.8	0.9	0	0
Bacteria(ml)	0.1	0.1	0.1	0.1	0.1	0.1	0.1	0	0.1

#### MIC Value Determination of Imipenem/Cilastatin Sodium Combined With Drug-Containing Serum

Nine sterile tubes were taken from each group; 1 mL broth was added to sterile tubes 1–7, 2 mL was added to tubes 8 and 9, and 1 mL Imipenem/cilastatin sodium solution was added to the 1^st^ tube (concentration of Imipenem/cilastatin sodium was 1,024 μg/mL). After the 1^st^ tube was mixed, 1 mL was taken out and added to the 2^nd^ tube, and after mixing in the 2^nd^ tube, 1 mL was taken out and added to the 3^rd^ tube, and the operation was repeated until the 7^th^ tube. After the 7^th^ tube was mixed, 1 mL was taken out and discarded. All of the pipetting operations were performed normatively in a biosafety cabinet using a 1,000 μL pipette specification.

Imipenem/cilastatin sodium group: 1 mL broth was added to each of the 1–7 tubes.

Imipenem/cilastatin sodium + drug-containing serum group: 0.4 mL of the drug-containing serum was added to each of the 1–7 tubes, and then the broth was added to make up the 2 mL system in the tubes.

The prepared 0.5 Mc concentration solution was diluted 10-fold, corresponding to a bacterial concentration of 1.5 x 10^7^ cfu/mL, and 0.1 mL was added to tubes 1–7 and 9. The 8^th^ tube only had broth as a negative control, and the 9^th^ tube had broth and bacterial solution as a model control. The solution in every tube was mixed well and placed in a 35°C incubator. The growth of bacteria was observed after 24 h. The test tube system of the Imipenem/cilastatin sodium + drug-containing serum groups is shown in **Table 3**.

**Table 3:** The test tube system of the Imipenem/cilastatin sodium + drug-containing serum groups.

System composition	Test tube number								
	1	2	3	4	5	6	7	8	9
Imipenem/cilastatin sodium(μg/ml)	256	128	64	32	16	8	4	0	0
drug-containing Serum(ml)	0.4	0.4	0.4	0.4	0.4	0.4	0.4	0	0
Added broth(ml)	0.6	0.6	0.6	0.6	0.6	0.6	0.6	–	–
Bacteria(ml)	0.1	0.1	0.1	0.1	0.1	0.1	0.1	0	0.1

### Ultrastructural Changes of MDR-PA Observed Under a Transmission Electron Microscope

Each group of tubes in the tube dilution method was cultured in a constant temperature and humidity incubator at 35°C for 24 h. With the test tube of Imipenem/cilastatin sodium MIC set as a standard, 3 mL PBS was added to the next test tube of each group, followed by centrifugation at 3,000 rpm for 10 min, after which the supernatant was discarded. The process was repeated three times to remove the serum, broth, antibiotics. Then the precipitate was fixed in glutaraldehyde, embedded, sectioned, and stained. The ultrastructure of the MDR-PA was observed by transmission electron microscopy and the nature and extent of the ultrastructural lesions were recorded.

## Results

### Growth Curve of MDR-PA

#### OD 570_nm_ Values of MDR-PA At Different Culture Time Points

The OD570nm values of the test tubes and negative control tubes of MDR-PA cultured for 24 h were measured. The results are shown in **Table 4**.

**Table 4:** MDR-PA OD570_nm_ value at different culture time points (1–24 h).

Time	OD_570nm_ value (*x* ± *s*)	Time	OD_570nm_ value (*x* ± *s*)	Time	OD_570nm_ value (*x* ± *s*)
1h	0.000 ± 0.000	9h	0.444 ± 0.005	17h	0.840 ± 0.004
2h	0.002 ± 0.001	10h	0.513 ± 0.043	18h	0.867 ± 0.015
3h	0.006 ± 0.002	11h	0.549 ± 0.026	19h	0.876 ± 0.007
4h	0.021 ± 0.003	12h	0.608 ± 0.021	20h	0.898 ± 0.014
5h	0.105 ± 0.010	13h	0.670 ± 0.020	21h	0.888 ± 0.004
6h	0.178 ± 0.012	14h	0.712 ± 0.014	22h	0.885 ± 0.009
7h	0.280 ± 0.008	15h	0.798 ± 0.007	23h	0.885 ± 0.003
8h	0.383 ± 0.010	16h	0.829 ± 0.027	24h	0.886 ± 0.007

The concentration of the bacterial solution was reflected by the OD 570_nm_ value. The higher the OD570_nm_ value, the higher the concentration of bacterial solution, indicating that the bacteria grew more. As seen in **Table 4**, within 24 h, with the prolongation of culture time, the OD570_nm_ value gradually increased, indicating that the MDR-PA in nutrient broth was growing and multiplying, and the amount of bacteria was gradually increasing. A negative control tube was set up in the test. The OD570_nm_ value in the table = OD570_nm_ value of the MDR-PA test tube - the OD570_nm_ value of the negative control tube, and the effect of the nutrient broth itself on the OD570_nm_ value was excluded.

#### MDR-PA Growth Curve Drawing

**Figure 2:**
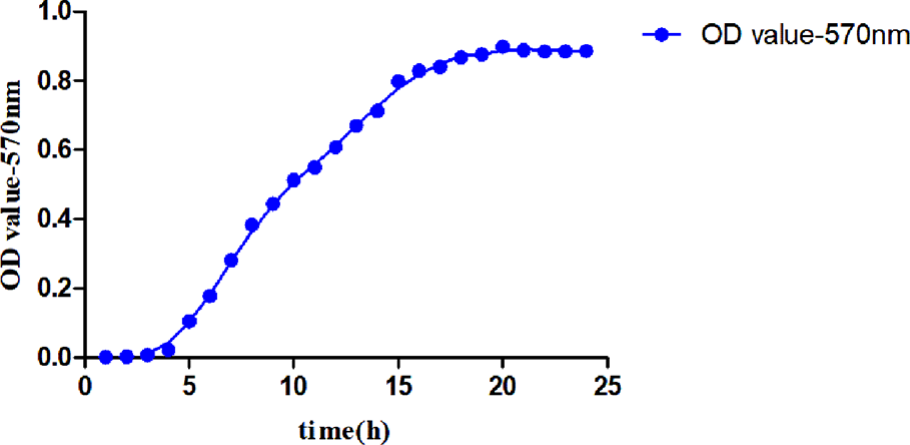
The growth curve of MDR-PA.

The culture time was taken as the abscissa and the OD570_nm_ value as the ordinate, the relationship between the concentration and the OD570_nm_ value of the MDR-PA at different time points is shown in **Figure 2**. The growth curve of the MDR-PA was plotted.

As shown in **Figure 2**, in the lag phase at 1–4 h, the bacteria entered a new nutrient environment. They rarely divided, and the number of bacteria did not increase. This stage was prepared for the next stage of bacterial division and proliferation. In the exponential growth phase at 5–20 h, the bacteria rapidly divided and proliferated in a two-division manner, increasing the number of viable cells exponentially. After 20 h, with the consumption of nutrients and the accumulation of metabolites, the number of bacterial proliferations decreased, the number of deaths gradually increased, and the bacteria entered a stable period. The proliferation and death of bacteria reached an equilibrium state, and the amount of bacteria remained at a certain level. During the susceptibility test, the optimal detection time of this MDR-PA strain was 16–20 h.

### MIC Determination of TCM Extract and Its Drug-Containing Serum by the Test Tube Dilution Method

FJHD Chinese medicine **e**xtract group: 1**^st^** tube 1 g/mL, 2^nd^ tube 0.8 g/mL, 3^rd^ tube 0.6 g/mL, 4^th^ tube 0.4 g/mL, 5^th^ tube 0.3 g/mL, 6^th^ tube 0.2 g/mL, 7^th^ tube 0.1 g/mL. Tubes 1–6 of the Chinese herbal extract group were clear and light yellow, and the 7^th^ tube was turbid, with bacterial growth. The model control tube was obviously turbid. The negative control tube was clear. The MIC of the Chinese medicine extract group was 0.2 g/mL. The test tube reaction system results of the Chinese medicine extract group are shown in **Table 5**.

**Table 5:** Test tube reaction system results of each simple Chinese medicine and drug-containing serum group.

Groups	Test tube number								
	1	2	3	4	5	6	7	8	9
Chinese medicine (g/ml)	1	0.8	0.6	0.4	0.3	0.2	0.1	0	0
Simple Chinese medicine	Clear	Clear	Clear	Clear	Clear	Clear	Turbid	Clear	Turbid
Drug-containing serum	Turbid	Turbid	Turbid	Turbid	Turbid	Turbid	Turbid	Clear	Turbid

Simple drug-containing serum groups: The 1–7^th^ tubes all showed varying degrees of turbidity and bacterial growth. The model control tube was obviously turbid. The negative control tube was clear. All of the simple drug-containing serum groups showed no obvious antibacterial effect. The test tube reaction system results of each drug-containing serum group are shown in **Table 5**.

The simple Imipenem/cilastatin sodium group included tubes 1–5, which were clear and light yellow. The 6^th^ tube appeared turbid. The 7^th^ tube was turbid. The model control tube was obviously turbid. The negative control tube was clear. The MIC of the simple Imipenem/cilastatin sodium group was 16 μg/mL.

In the Imipenem/cilastatin sodium + blank serum group, tubes 1–5 were clear and light yellow. The 6^th^ tube appeared turbid. The 7^th^ tube was turbid. The model control tube was obviously turbid. The negative control tube was clear. The MIC of the Imipenem/cilastatin sodium + blank serum group was 16 μg/mL.

In the Imipenem/cilastatin sodium + 0.5-fold drug-containing serum group, tubes 1–5 were clear and light yellow. The 6^th^ tube appeared turbid. The 7^th^ tube was turbid. The model control tube was obviously turbid. The negative control tube was clear. The MIC of Imipenem/cilastatin sodium + 0.5-fold drug-containing serum group was 16 μg/mL.

In the Imipenem/cilastatin sodium + 1-fold drug-containing serum group, tubes 1–5 were clear and light yellow. The 6^th^ tube appeared turbid. The 7^th^ tube was turbid. The model control tube was obviously turbid. The negative control tube was clear. The MIC of Imipenem/cilastatin sodium + 1-fold drug-containing serum group was 16 μg/mL.

In the Imipenem/cilastatin sodium + 2-fold drug-containing serum group, tubes 1–5 were clear and light yellow. The 6^th^ tube appeared turbid. The 7**^th^** tube was turbid. The model control tube was obviously turbid. The negative control tube was clear. The MIC of the Imipenem/cilastatin sodium + 2-fold drug-containing serum group was 16 μg/mL.

In the Imipenem/cilastatin sodium + 4-fold drug-containing serum group, tubes 1–6 were clear and light yellow. The 7**^th^** tube appeared turbid. The model control tube was obviously turbid. The negative control tube was clear. The MIC of Imipenem/cilastatin sodium + 4-fold drug-containing serum group was 8 μg/mL.

In the Imipenem/cilastatin sodium + 8-fold drug-containing serum group, tubes 1–6 were clear and light yellow. The 7^th^ tube appeared turbid. The model control tube was obviously turbid. The negative control tube was clear. The MIC of Imipenem/cilastatin sodium + 8-fold drug-containing serum group was 8 μg/mL. The test tube reaction system results of each Imipenem/cilastatin sodium+ drug-containing serum group are shown in **Table 6**.

**Table 6:** Test tube reaction system results of each Imipenem/cilastatin sodium + drug-containing serum group.

Groups	Test tube number								
	1	2	3	4	5	6	7	8	9
Imipenem/cilastatin sodium (μg/ml)	256	128	64	32	16	8	4	0	0
Simple Imipenem/cilastatin sodium	Clear	Clear	Clear	Clear	Clear	Turbid	Turbid	Clear	turbid
Imipenem/cilastatin sodium + blank serum	Clear	Clear	Clear	Clear	Clear	Turbid	Turbid	Clear	turbid
Imipenem/cilastatin sodium + 0.5-fold	Clear	Clear	Clear	Clear	Clear	Turbid	Turbid	Clear	turbid
Imipenem/cilastatin sodium + 1-fold	Clear	Clear	Clear	Clear	Clear	Turbid	Turbid	Clear	turbid
Imipenem/cilastatin sodium + 2-fold	Clear	Clear	Clear	Clear	Clear	Turbid	Turbid	Clear	turbid
Imipenem/cilastatin sodium + 4-fold	Clear	Clear	Clear	Clear	Clear	Clear	Turbid	Clear	turbid
Imipenem/cilastatin sodium + 8-fold	Clear	Clear	Clear	Clear	Clear	Clear	Turbid	Clear	turbid

### Morphological Changes of MDR-PA Observed by Transmission Electron Microscopy

In the test tube dilution method, the MIC value of Imipenem/cilastatin sodium was 16 μg/mL in the 5^th^ tube. According to the results of the test tube dilution method, observation specimens were prepared with test tube reaction system after the 6^th^ tube of each group. The model control group was obtained by culturing MDR-PA in pure broth, using the tube dilution method.

#### Morphological Changes of MDR-PA

In the model control group, MDR-PA cells growing in pure broth showed characteristic shapes of Gram-negative bacteria, with uniform electron density and a clear extracellular outer membrane and intracellular protoplast.

In the simple Imipenem/cilastatin sodium group, the outer membrane was damaged and blurred in the polar region of MDR-PA cells. The inner and outer membrane integrity was damaged. Aggregated dense particles were observed in the protoplast. The cellular inclusions disappeared but outer membranes remained in some cells, exhibiting an empty shell state.

In the Imipenem/cilastatin sodium + 0.5-fold drug-containing serum group, the outer membrane was damaged in the polar region of MDR-PA cells, and part of the outer membrane ruptured. Aggregated dense particles were observed in the protoplast. The cellular inclusions disappeared but the outer membranes remained in some cells. The cell surfaces showed vacuoles. The inner and outer membrane integrity was damaged, and the two membrane structures contained cytoplasm, which protruded from the surface of the bacteria.

In the Imipenem/cilastatin sodium + 1-fold drug-containing serum group, the inner and outer membrane were damaged.

In the Imipenem/cilastatin sodium + 2-fold drug-containing serum group, the outer membrane was damaged and partially ruptured. Aggregated dense particles and vacuoles were observed in the protoplast. Cellular inclusions totally disappeared in some cells. Cell surfaces showed vesicles that appeared as rough structural spots that could have been cellular constituents leaking from cells.

In the Imipenem/cilastatin sodium + 4-fold drug-containing serum group, the outer membrane was damaged in the polar region and partially ruptured. Aggregated dense particles were observed in the protoplast. Cellular inclusions totally disappeared in some cells. The inner and outer membrane integrity was damaged.

**Figure 3:**
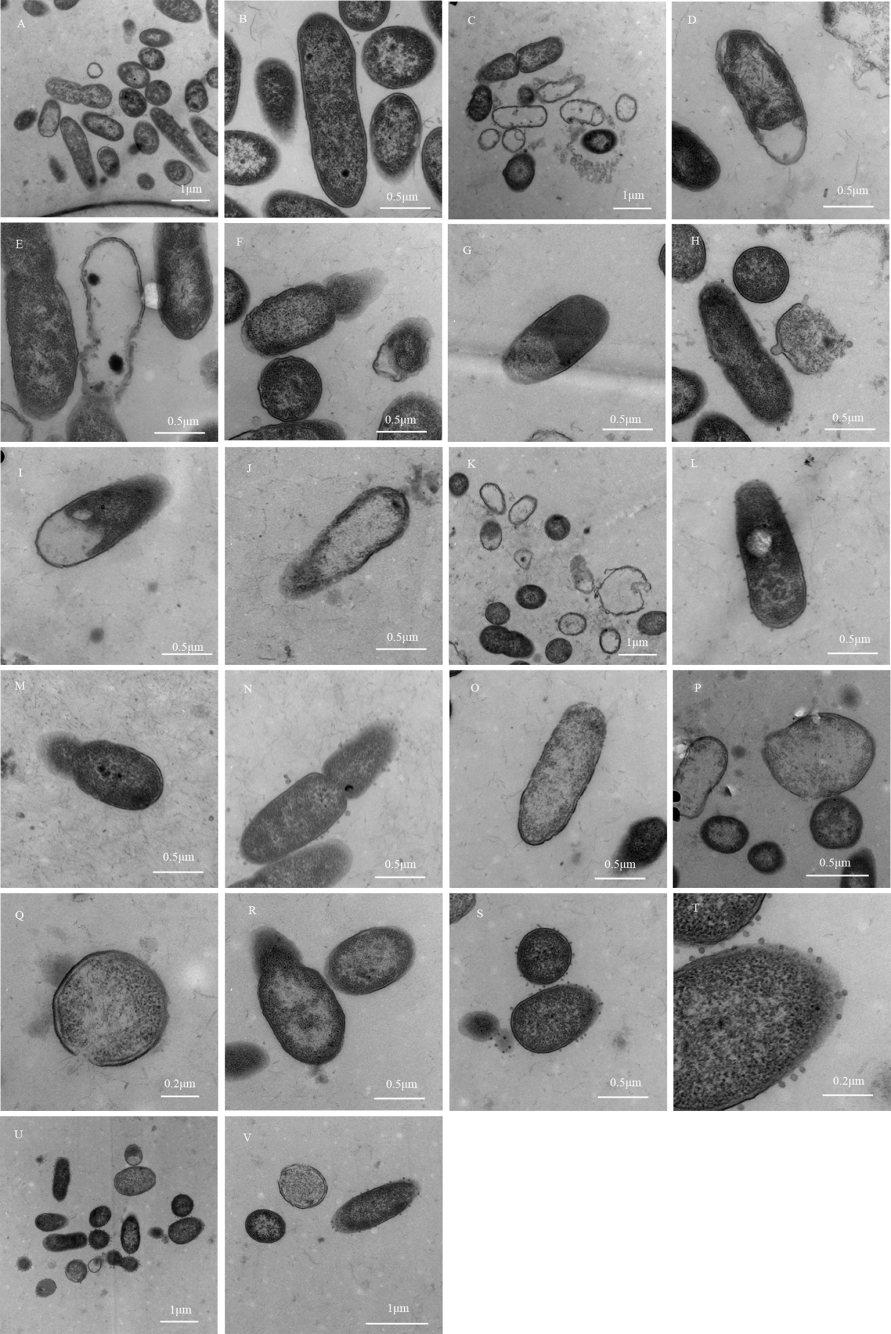
Morphological changes of MDR-PA. **(A, B)**: Model control group; **(C–F)**: Simple Imipenem/cilastatin sodium group; **(G, H)**: Imipenem/cilastatin sodium + 0.5-fold drug-containing serum group; **(I, J)**: Imipenem/cilastatin sodium + 1-fold drug-containing serum group; **(K–N)**: Imipenem/cilastatin sodium + 2-fold drug-containing serum group; **(O–R)**: Imipenem/cilastatin sodium + 4-fold drug-containing serum group; **(S–V)**: Imipenem/cilastatin sodium + 8-fold drug-containing serum group.

In the Imipenem/cilastatin sodium + 8-fold drug-containing serum group, the outer membrane was damaged in the polar region and partially ruptured. Aggregated dense particles were observed in the protoplast. The inner and outer membrane integrity was damaged. Cell surfaces showed vesicles. The cells were severely damaged. The Morphological changes of MDR-PA in all of the groups are shown in **Figure 3**.

#### Statistical Results of MDR-PA Electron Microscopy

According to the photomicrograph of the electron micrograph, Imipenem/cilastatin sodium + 0.5-fold, 1-fold, and 2-fold drug-containing serum groups showed bacterial structure damage, similar to the simple Imipenem/cilastatin sodium group, and there was no significant difference. The degree of bacterial destruction was more obvious in the Imipenem/cilastatin sodium + 4-fold and 8-fold drug-containing serum groups than in the simple Imipenem/cilastatin sodium group, with an increasing trend. The region where the bacteria distribution density was relatively uniform under 30,000 times of field of view was selected and locked. In the region the total number of visible bacteria, the number of damaged bacteria and the bacterial damage ratio was counted. The statistical results of the model control group (**Figure 3B**), the simple Imipenem/cilastatin sodium group (**Figure 3C**), and the Imipenem/cilastatin sodium + 8-fold drug-containing serum group (**Figure 3V**) are listed in **Table 7**.

**Table 7:** Bacterial damage ratio of the three groups.

Bacterial grouping	Damaged bacteria	Visible bacteria	Damage ratio	Fisher exact test	
				F	P
Model control	2	15	13%	11.199	0.003
simple Imipenem/cilastatin sodium group	6	12	50%		
Imipenem/cilastatin sodium + 8-fold	11	15	73%		

Using the SPSS 19.0 statistical software Fisher's exact test, F = 11.199, P = 0.003, less than 0.05. The difference was statistically significant, and the antibacterial effect of Imipenem/cilastatin sodium + 8-fold drug-containing serum group was significantly better than that of the simple Imipenem/cilastatin sodium group.

## Discussion

In a previous clinical study (Xu et al., 2018), our research group confirmed that FJHD full active ingredient extract formula granules significantly improved the effective rate with a course of treatment for more than 2 weeks in the elderly patients with pneumonia. Compared with simple antibiotics, FJHD combined with antibiotics markedly decreased the expectoration score during the first week(P < 0.05), improvement in expectoration was conducive to airway patency. During the second week, wheezing, shortness of breath, and other symptoms were also significantly improved. During the third week, body temperature was stable. FJHD improved lesion absorption with Pneumonia Severity Index (PSI) class II (P < 0.05) and SMART-COP score 1 (P < 0.05). FJHD significantly decreased the arterial carbon dioxide partial pressure after a 1-week treatment. During treatment, there were no serious adverse events in FJHD combined with antibiotics, which were safe and reliable and could be widely used in general hospitals and primary hospitals. The reason why FJHD has the above curative effect in clinic is preliminarily explored in this experiment. The inhibition mechanism on MDR-PA *in vitro* is analyzed and discussed as follows.

### Significance of Bacterial Growth Curve

Bacterial growth generally went through four stages, namely, the lag phase, exponential growth phase, plateau phase, and decay phase (Peng et al., 2010), which were reflected in the growth curve of the bacteria. In the exponential growth phase, bacterial morphology, staining, and physiological activity were typical and sensitive to the effects of external environmental factors. This phase was an ideal stage to study bacterial biological characteristics and drug sensitivity tests. The optimal detection time for drug sensitivity test of MDR-PA was 16–20 h, which was consistent with the cultivation time for dilution drug sensitivity test of PA in the implementation standard of antimicrobial susceptibility test (CLSI, 2014) formulated by CLSI. In the case of *in vitro* susceptibility testing and bacterial morphological observation, the results of bacteria cultured at this stage could be more reliable. The length of each period of bacterial growth was related to the characteristics of different strains, medium composition, and culture conditions (Zhou and Ye, 2013). Therefore, in the study of bacterial experiments, understanding the proliferation of experimental bacteria and drawing growth curves was a very important preliminary basis for drug sensitivity experiments.

The bacterial growth curve could dynamically and intuitively reflect the growth and proliferation process and reveal the growth law of bacteria. In the susceptibility test of TCM *in vitro*, the MIC could only determine whether it had any effect on bacteria, whereas the growth curve reflected the interaction between the TCM and bacteria, objectively revealing the effect of TCM on bacterial growth and proliferation. By studying the effect of TCM on the growth curve of bacteria, we found the effects of drugs on the growth and proliferation process of the bacterial population, and we obtained a better understanding of the adaptation degree of bacteria to the nutrient environment, the utilization of medium components, the different bacterial effects of different components in the drug and a variety of meaningful information, such as the strongest and weakest time of Pharmacodynamic effect (Liu et al., 2007; Huang et al., 2017).

The growth curve of the bacteria had high application value in the *in vitro* drug sensitivity test. In the susceptibility test, it was necessary to draw a growth curve exclusive to the experimental bacteria, and it was also the preliminary basis for the susceptibility test. TCM had good effects on delaying, inhibiting, and even reversing bacterial resistance. The bacterial growth curve could directly reflect the growth and proliferation of bacteria during the interaction between drugs and bacteria, and the direct effect of drugs on bacterial growth could be found. In light of the world's toughest medical problems, such as current refractory infectious disease caused by drug-resistant bacteria infections, it is of non-negligible social and economic value in screening of TCMs with antibacterial effects and their effective components by *in vitro* susceptibility test and studying on the mechanism of TCM on resisting drug-resistant bacteria, thereby reducing the use of antibiotics and protecting antibiotics.

### Exploration of Drug-Containing Serum System

In the Imipenem/cilastatin sodium + 0.5-fold, 1-fold, 2-fold, 4-fold, and 8-fold drug-containing serum groups, the amount of serum added was 0.8 mL, 0.6 mL, and 0.4 mL, respectively. The results showed that when Imipenem/cilastatin sodium was combined with 0.4 mL drug-containing serum, the test tube was clearer and the antibacterial effect was better. Imipenem/cilastatin sodium group MIC was in the 5^th^ tube. The 6^th^ tube of Imipenem/cilastatin sodium + 4-fold and 8-fold drug-containing serum groups were clearer than the 6th tube of Imipenem/cilastatin sodium group. In the 6^th^ tube of each group, Imipenem/cilastatin sodium + 8-fold serum (brightness) was greater than Imipenem/cilastatin sodium + 4-fold serum and greater than Imipenem/cilastatin sodium + 2-fold serum and greater than the Imipenem/cilastatin sodium group. A suitable system for drug-containing serum was 20%. In the *in vitro* experimental study of TCM serum, the amount of serum added was an important factor affecting the experimental results. Therefore, it was of great significance to explore the optimal reaction system in the experiment. In the preliminary experiment, the research group tried 40% (0.8 mL), 30% (0.6 mL), and 20% (0.4 mL) reaction system. As a result, in the 20% reaction system, the antibacterial effect of Imipenem/cilastatin sodium combined with drug-containing serum was the best. According to the relevant literature, there was no clear regulation on the concentration of drug-containing serum about *in vitro* experiments. Most of the drug-containing serum accounted for 5% to 20% of the reaction system (Huang et al., 2011; Chen et al., 2014). Some scholars believed that considering the serum tolerance of the cells cultured in the experimental system, the amount of drug-containing serum should not exceed 20% (Pan et al., 2002; Wang et al., 2006). This might be related to the fact that serum as a “nutrient” affected the reproduction of bacteria when the proportion of drug-containing serum increased.

### TCM Combined With Antibiotics Enhances the Antibacterial Effect of Antibiotics

Under the condition that model control tube bacteria grow normally and the negative control tube grow aseptically, the minimum concentration of reagent in the test tube without turbidity was the MIC of the reagent. The MIC of the Chinese herbal extract group was in the 6^th^ tube, and the MIC value was 0.2 g/mL. The Imipenem/cilastatin sodium group MIC was in the 5^th^ tube and the MIC value was 16 μg/mL. The MIC values of Imipenem/cilastatin sodium + blank serum and Imipenem/cilastatin sodium + 0.5 times, 1-fold and 2-fold drug-containing serum groups were still 16 μg/mL.The Imipenem/cilastatin sodium + 4-fold and 8-fold drug-containing serum groups MIC were in the 5^th^ tube and their MIC values were 16 μg/mL. This experiment found that the Imipenem/cilastatin sodium + 4-fold and 8-fold drug-containing serum could reduce the MIC value of Imipenem/cilastatin sodium by a gradient. This might be related to the drug-containing serum inhibiting the drug resistance of MDR-PA, thereby increasing the antibacterial potency of the antibiotics with weak antibacterial activity against MDR-PA. TCM combined with antibiotics can enhance the bacteriostatic effect of antibiotics.

### Electron Microscopy Results

In this experiment, it was found that when the Imipenem/cilastatin sodium concentration was consistent in each Imipenem/cilastatin sodium combined with drug-containing serum group (8 μg/mL), as the fold of equivalent dose drug-containing serum increased, the peripheral secretory granules of bacteria increased. Under transmission electron microscopy, the model control group and simple Imipenem/cilastatin sodium group showed no secretory granules at bacterial periphery. Imipenem/cilastatin sodium + 0.5-fold drug-containing serum group showed individual granules, and Imipenem/cilastatin sodium + 2-fold drug-containing serum group showed a few particles. Imipenem/cilastatin sodium + 8-fold drug-containing serum group showed more exocrine particles in the periphery of the bacteria. The production of exocrine granules of Gram-negative bacteria was considered to be a way for live bacteria to release endotoxin (Gao et al., 1997). The endotoxin released by Gram-negative bacilli was a macromolecular colloidal substance. Its chemical structure was stable, not easy to be destroyed and eliminated, and it stayed longer in the body and could continuously stimulate lymphocytes (Gong, 2014) to induce an immune response.

Therefore, this experiment demonstrated that, when the drug-containing serum combined with antibiotics acted on MDR-PA, in addition to directly destroying the bacterial structure, causing damage and lysis, the active ingredients in the drug-containing serum could also release endotoxin from the bacteria to stimulate lymphocytes and produce an immune response that could clear bacteria.

## Prospects

This experiment initially showed that the antibacterial effect of TCM combined with antibiotics was stronger than simple antibiotics against multidrug-resistant pathogens. However, the interventional effect of TCM on drug-resistant mechanism of pathogens has not yet been deeply explored. The intervention mechanism of TCM against bacterial resistance should be further studied, and the active ingredients of TCM should be further revealed to provide a more reliable experimental basis for the prevention and treatment of respiratory drug-resistant infectious diseases.

## Data Availability Statement

The datasets generated for this study are available on request to the corresponding authors.

## Ethics Statement

The animal study was reviewed and approved by the Beijing University of Chinese Medicine Medical and Experimental Animal Ethics Committee.

## Author Contributions

HX and CW provided detailed guidance on the implementation of this experiment. HX wrote, revised, and improved the paper. CL and MW participated in the experiment and edited the manuscript. GL, JM, LL, MeC, SZ, and YW performed the experiments. ML, HW, MiC, XY, and YL participated in part of the experiments.

## Funding

This work was funded by the Research Fund for the Doctoral Program of Higher Education of China (No. 20130013120004), the National Natural Science Foundation of China (Nos. 81473661 and 81573924), and Dongzhimen Hospital Affiliated to Beijing University of Chinese Medicine Qingmiao Talent Program (No. DZMYS-201608).

## Conflict of Interest

The authors declare that the research was conducted in the absence of any commercial or financial relationships that could be construed as a potential conflict of interest.
