# Supplementary material for: In Vitro Antibacterial Experiment of Fuzheng Jiedu Huayu Decoction Against Multidrug-Resistant Pseudomonas aeruginosa
Source: Front Pharmacol. 2020 Feb 12;10:1682. doi: 10.3389/fphar.2019.01682 (PMC7029191; doi:10.3389/fphar.2019.01682)
Supplement: Supplementary file 1 [file DataSheet_1.pdf]

UHPLC-Q-Orbitrap HRMS analysis

The UHPLC separation was carried out on an UltiMate 3000 liquid chromatograph equipped with a quaternary pump, an online degasser, a thermostatic column compartment (Thermo Scientific, Sunnyvale, CA, USA), and a PAL autosampler (CTC analytics, Zwingen, Switzerland). A Waters ACQUITY BEH C_{18} analytical column (100 mm × 2.1 mm, 1.7 μm) was employed, and the column temperature was kept at 35 ℃ during use. An optimized gradient was used at a constant flow rate of 0.3 $mL·min^{-1}$ using Milli-Q water with 0.05% formic acid (Solvent A) and acetonitrile (Solvent B). The initial conditions were set at 40% B, and held for 1 min. Then, the gradient was programmed to 100% B at 7 min, and kept for 1 min. Finally, the gradient was returned to the initial conditions at 8.1 min, and then re-equilibrated for 1 min to complete the whole run. A sampling volume of 3 μL was injected for each run with a 200 μL needle wash solvent of 90% aqueous methanol solution.

The UHPLC system was used in conjunction with a benchtop Q Exactive hybrid quadrupole-Orbitrap mass spectrometer (Thermo Scientific, Bremen, Germany). The ionization of target analytes was enabled via a heated electrospray ionization (HESI) source operated in ESI^- mode with the following parameters: capillary temperature of 320 ℃; auxiliary gas heater temperature of 300 ℃; sheath gas, auxiliary gas, and sweep gas flow rates of 40, 10, and 1 (in arbitrary units); spray voltages of 2.9 kV. Full-scan data within the mass range of mass-to-charge ratio (*m/z*) 100–1000 was acquired at a mass resolution of 70,000 (full width at half maximum (FWHM) defined for *m/z* 200 at a scan rate of 3 Hz).

.
